# Patient and hospital characteristics associated with do-not-resuscitate/do-not-intubate orders: a cross-sectional study based on the Taiwan stroke registry

**DOI:** 10.1186/s12904-023-01257-7

**Published:** 2023-09-15

**Authors:** Hsu-Ling Yeh, Fang-I Hsieh, Li-Ming Lien, Wen-Hua Kuo, Jiann-Shing Jeng, Yu Sun, Cheng-Yu Wei, Po-Yen Yeh, Hei-Tung Yip, Cheng-Li Lin, Nicole Huang, the Taiwan Stroke Registry Investigators, Kai-Cheng Hsu

**Affiliations:** 1 Institute of Public Health, National Yang Ming Chiao Tung University, Taipei, Taiwan; 2 Department of Neurology, Shin Kong Wu Ho-Su Memorial Hospital, Taipei, Taiwan; 3 School of Public Health, College of Public Health, Taipei Medical University, Taipei, Taiwan; 4 Institute of Science, Technology, and Society, National Yang Ming Chiao Tung University, Taipei, Taiwan; 5 Stroke Center, Department of Neurology, National Taiwan University Hospital, Taipei, Taiwan; 6 Department of Neurology, En Chu Kong Hospital, New Taipei City, Taiwan; 7 Department of Exercise and Health Promotion, College of Kinesiology and Health, Chinese Culture University, Taipei, Taiwan; 8 Department of Neurology, St. Martin de Porres Hospital, Chiayi City, Taiwan; 9 Management office for Health Data, China Medical University Hospital, Taichung, Taiwan; 10 Department of Public Health, China Medical University, Taichung, Taiwan; 11 Institute of Hospital and Health Care Administration, National Yang Ming Chiao Tung University, No. 155, Section 2, Li-Nong Street, Taipei 112, Taipei, Taiwan; 12 Department of Neurology, China Medical University Hospital, Taichung, Taiwan

**Keywords:** Resuscitation orders, Stroke, Terminal care, Inpatients, East Asia

## Abstract

**Background:**

Previous studies of do-not-resuscitate (DNR) or do-not-intubate (DNI) orders in stroke patients have primarily been conducted in North America or Europe. However, characteristics associated with DNR/DNI orders in stroke patients in Asia have not been reported.

**Methods:**

Based on the Taiwan Stroke Registry, this nationwide cross-sectional study enrolled hospitalized stroke patients from 64 hospitals between 2006 and 2020. We identified characteristics associated with DNR/DNI orders using a two-level random effects model.

**Results:**

Among the 114,825 patients, 5531 (4.82%) had DNR/DNI orders. Patients with acute ischemic stroke (AIS) had the highest likelihood of having DNR/DNI orders (adjusted odds ratio [aOR] 1.76, 95% confidence interval [CI] 1.61–1.93), followed by patients with intracerebral hemorrhage (ICH), and patients with subarachnoid hemorrhage (SAH) had the lowest likelihood (aOR 0.53, 95% CI 0.43–0.66). From 2006 to 2020, DNR/DNI orders increased in all three types of stroke. In patients with AIS, women were significantly more likely to have DNR/DNI orders (aOR 1.23, 95% CI 1.15–1.32), while patients who received intravenous alteplase had a lower likelihood (aOR 0.74, 95% CI 0.65–0.84). Patients with AIS who were cared for by religious hospitals (aOR 0.55, 95% CI 0.35–0.87) and patients with SAH who were cared for by medical centers (aOR 0.40, 95% CI 0.17–0.96) were significantly less likely to have DNR/DNI orders.

**Conclusions:**

In Taiwan, DNR/DNI orders increased in stroke patients between 2006 and 2020. Hospital characteristics were found to play a significant role in the use of DNR/DNI orders.

**Supplementary Information:**

The online version contains supplementary material available at 10.1186/s12904-023-01257-7.

## Background

Stroke is a leading cause of death and disability worldwide. Japan and Taiwan report the highest incidence rates of stroke in Asia. Although stroke-related mortality rates are decreasing in East Asia, many stroke survivors experience lifelong disabilities and poor quality of life [1]. These individuals require timely access to palliative care. However, guidelines or laws requiring professional judgment regarding patients’ terminal status before administering palliative care may be a serious obstacle in these countries [2].

Strictly speaking, do-not-resuscitate (DNR) only refers to the limitation of cardiopulmonary resuscitation (CPR) in the event of cardiac arrest [3]. However, in practice, a DNR order is often followed by a limitation of other types of care. For instance, a do-not-intubate (DNI) order may be issued, which withholds intubation and mechanical ventilation (IMV) in cases of pre-arrest respiratory failure [4, 5]. In some cases, patients with DNR orders may also refuse surgical interventions [6]. It is worth noting that signing a DNR consent form often marks the beginning of palliative care [5, 7].

In 2000, Taiwan became the first country in Asia to pass legislation specifically addressing end-of-life and palliative care, with the Hospice Palliative Care Act [8]. In Japan, the Guideline for Medical Decision-Making Process in End-of-Life Care, issued in 2007, guides the use of DNR orders, but relevant laws have not been passed [2]. In South Korea, the “Act on Decisions on Life-Sustaining Treatment for Patients in Hospice and Palliative Care or at the End of Life” was passed in 2016, and fully enforced in 2018. This act limits the “terminal stage” to patients with acquired immune deficiency syndrome, chronic liver cirrhosis, chronic obstructive pulmonary disease, cancer, and other specified diseases, but stroke is not included [2, 9]. There have been no studies of end-of-life care in patients with stroke in Korea or Japan yet in English literature [2, 9, 10]. Most studies have been carried out in North America [11–18] or Europe [5, 19–21]. Several of the few studies published in Asia were in Taiwan [22–24].

Although withholding and withdrawing life-sustaining treatment (LST) are considered morally equivalent in Western countries, this is generally not the case in Asia [2]. In a 2012 survey of intensive care unit physicians, 80% of physicians in Taiwan and 79% in Korea reported that withholding and withdrawing LST were ethically different [2, 25]. Specifically, more than 80% of physicians from Taiwan tended to withhold but not withdraw therapies, while less than 3% responded that they often withdrew LST [25]. Despite the legal permissibility of withdrawing LST after the amendments of the Hospice Palliative Care Act in 2011 and 2013, many physicians in Taiwan still hesitate to do so.

In Taiwan, under the Hospice Palliative Care Act, doctors can only forgo LST, such as IMV, CPR, or resuscitation drugs, when terminal patients or their families sign formal written consents. The formal written consent form used in Taiwan is a combined form of DNR and DNI, despite being called a “do not resuscitate” consent. The right to refuse CPR/IMV is only entitled to terminal patients, defined as individuals with incurable diseases who cannot avoid death in the near future based on medical evidence [2]. Thus, end-of-life and palliative care have been overlooked in patients with non-cancer diseases such as stroke. End-of-life care often begins late in the clinical course when stroke patients have already lost capacity and are under IMV [26]. In two previous studies of stroke patients in Taiwan, DNR consents were all signed by surrogates [22, 23]. However, with the progress of the palliative care movement globally and domestically, the National Health Insurance program in Taiwan has begun reimbursing palliative care for eight types of non-cancer diseases, including severe stroke, since 2009. While this change in the reimbursement policy does not amend the legal definition of terminal patients, it may serve as an official green light for some physicians and hospitals to diagnose severe stroke patients as terminal and issue DNR or DNI orders after obtaining consent.

Studies on factors related to end-of-life care in patients with stroke have mainly been conducted in Western countries [16, 27–31]. Age, sex, ethnicity, illness severity, and comorbidities are significant factors [16, 29, 30]. In a qualitative study, hospital culture and policy were found to influence physicians’ attitudes toward patient autonomy in DNR decision-making [32]. Previous studies have attempted to investigate differences in the frequency of DNR orders [27], withdrawal of care [31], and palliative care encounter [28] among various hospital types in patients with stroke, but no similar study has been conducted in East Asia [2, 33]. Therefore, we investigated a wide array of patient and hospital factors associated with DNR/DNI orders among stroke patients in Taiwan. We hypothesized that the use of DNR without IMV is increasing and that variations in DNR use exist between different types of hospitals, as hospital cultures and policies may affect the timing of considering DNR orders and interpretations of the definition of terminal patients [32].

## Methods

### Study design and data source

This cross-sectional study is based on the Taiwan Stroke Registry (TSR), a nationwide hospital-based registry that has been enrolling stroke patients since 2006. The TSR includes 19 academic medical centers, 37 regional hospitals, and 8 district hospitals. Patients admitted within ten days after stroke onset are included, and the data is collected prospectively by neurologists and study nurses trained by the TSR. The type of stroke is diagnosed by the treating neurologist or neurosurgeon, and then double-confirmed by another neurologist who reviews the neuro-images and fills in the registration form accordingly. Patients with traumatic types of intracerebral hemorrhage (ICH) or subarachnoid hemorrhage (SAH) are excluded from the registry. The registry has enrolled more than 150,000 patients to date. The details have been described in previous studies [34, 35].

### Study population

**Fig. 1:**
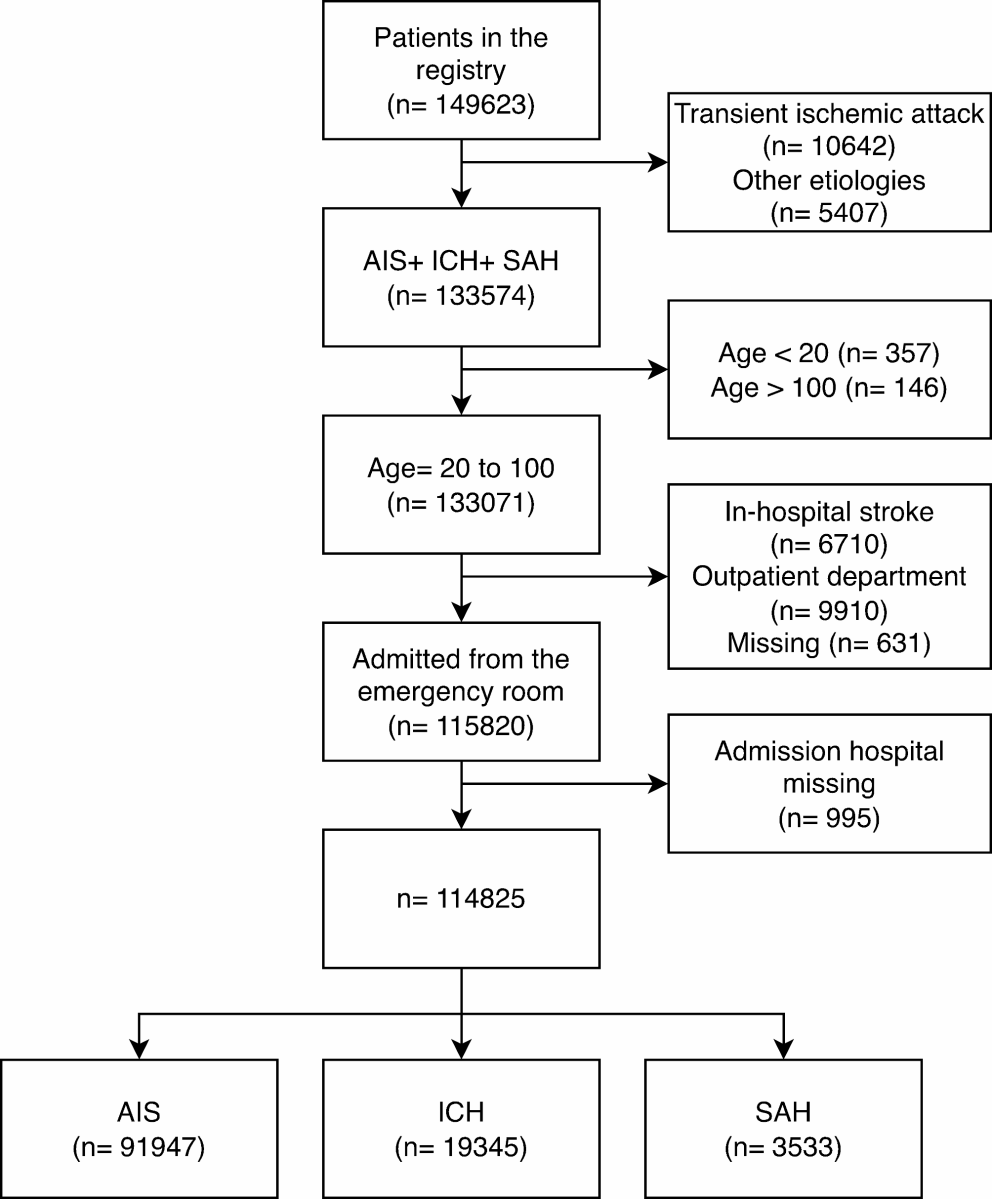
Flowchart of patient selection. AIS, acute ischemic stroke; ICH, intracerebral hemorrhage; SAH, subarachnoid hemorrhage

We included patients aged 20 to 100 admitted through the emergency room between 2006 and 2020 with acute ischemic stroke (AIS), ICH, or SAH. Patients were excluded if their admission source data were missing (Fig. 1).

### Outcomes

Since DNR orders may be issued after aggressive treatments fail, previous studies have used various definitions to distinguish early DNR orders from late DNR orders (i.e. DNR orders issued after intensive treatment fails) [12, 13, 36]. In Taiwan, the official “do not resuscitate” consent form is actually a combined form for DNR and DNI. It is common practice to withhold IMV after a do-not-resuscitate consent is signed. Therefore, if a DNR consent is signed before intubation, the patient would not be intubated, and this DNR order would be classified as a DNR without IMV (early DNR). If a DNR consent is signed after the failure of aggressive treatment, including IMV, this DNR order would be classified as a DNR with IMV (late DNR). DNR orders for patients who did not receive IMV during their hospitalization were defined as DNR/DNI orders, which were used as the primary outcome to capture DNR orders signed early in the clinical course.

### Characteristics

Covariates included age, sex, National Institute of Health Stroke Scale (NIHSS) scores upon admission [37, 38], the combined score of eye and motor tests on the Glasgow Coma Scale (GCS E + M) upon admission [5, 39, 40], whether patients with AIS received intravenous alteplase, and comorbidities including histories of uremia, congestive heart failure, cancer, and previous stroke. The diagnoses of comorbidities recorded in the Taiwan Stroke Registry are reported by professional study nurses based on patient’s medical records. Congestive heart failure is defined based on the clinical diagnosis made by cardiologists or the treating physician in patient’s medical records. Uremia is defined as end-stage renal disease when dialysis is indicated, as suggested by nephrologists. The physicians do certainly take laboratory data into consideration while making their diagnoses, but do not make the diagnoses based on laboratory data alone. Hospital characteristics such as accreditation level, religious affiliation, and ownership were also included. The number of years from 2006 to admission was also calculated and included in the models as “years-from-2006.”

### Statistical analysis

Continuous variables were expressed as mean with standard deviation. Ordinal variables were expressed as median with interquartile range. Count and percentage were used for categorical variables. To assess baseline characteristics between patients with and without DNR/DNI orders, the Student’s t-test was used for continuous variables, the Wilcoxon (Mann-Whitney) rank-sum test was used for ordinal variables, and the chi-square test was used for categorical variables.

The variables in the models were chosen based on their potential confounding effects in the causal diagram [41]. Age was centered to the mean and NIHSS was centered to the median. Missing data for age, NIHSS, GCS (E + M), sex, uremia, congestive heart failure, cancer, and previous stroke were imputed using multiple imputation for each of the three types of stroke. Covariates included centered age, centered NIHSS, GCS (E + M), sex, comorbidities (uremia, congestive heart failure, cancer, and previous stroke), years-from-2006, and hospital characteristics (accreditation level, religious affiliation, and ownership). In patients with AIS, receiving intravenous alteplase was an additional variable. A two-level random effects model was used to evaluate independent associations with DNR/DNI orders in patients with AIS, ICH, or SAH collectively and respectively. Forest plots were used to display the adjusted odds ratios (aOR) with 95% confidence intervals (CI). All analyses were performed using Stata software (version 14.0, StataCorp LP, USA). The significance level was set at a *P* value of less than 0.05. Subgroup analyses of AIS, ICH, and SAH were planned in advance, and this study was exploratory in nature, so the significance level was not adjusted for multiple comparisons [42–44].

## Results

A total of 114,825 patients were enrolled (Fig. 1), and 4.82% (5531) received DNR/DNI orders. Among the 91,947 patients with AIS, 4.9% (4464) had DNR/DNI orders, and 26.3% of these patients with DNR/DNI orders (1173/4464) died in the hospital. For the 19,345 ICH patients, 4.9% (954) had DNR/DNI orders, and 36.4% (347/954) died in the hospital. Among the 3533 patients with SAH, 3.2% (113) received DNR/DNI orders, and 48.7% (55/113) died in the hospital.

The DNR/DNI group were older, had a higher proportion of females, had higher NIHSS scores, lower GCS scores, a higher proportion of comorbidities, and a higher proportion of previous stroke (Table 1). The percentage of patients with AIS who had DNR/DNI orders was 2.02% in 2006, 6.98% in 2019, and 9.60% in 2020. For patients with ICH, the corresponding percentages were 2.93% in 2006, 9.23% in 2019, and 12.41% in 2020. For patients with SAH, the percentages were 2.99% in 2006, 3.45% in 2019, and 14.63% in 2020.

**Table 1:** Patient and hospital characteristics of study participants

Characteristic	DNR/DNI		*P*
	No (n = 109,294)	Yes (n = 5531)	
Stroke type			< 0.001
AIS	87,483 (80.04)	4464 (80.71)	
ICH	18,391 (16.83)	954 (17.25)	
SAH	3420 (3.13)	113 (2.04)	
Age (Years)^*^	67.1 ± 13.5	77.8 ± 12.3	< 0.001
NIHSS^†^	5 (3–12)	20 (11–27)	< 0.001
GCS (E + M)^†^	10 (10–10)	8 (6–10)	< 0.001
Female	42,122 (38.54)	2967 (53.64)	< 0.001
Uremia	3671 (3.36)	356 (6.44)	< 0.001
Congestive heart failure	2691 (2.46)	523 (9.46)	< 0.001
Cancer	4335 (3.97)	675 (12.20)	< 0.001
Previous stroke	29,012 (26.54)	1933 (34.95)	< 0.001
Years-from-2006^*^	6.1 ± 3.7	7.7 ± 3.6	< 0.001
Medical center	67,043 (61.34)	3541 (64.02)	< 0.001
Religious hospital	14,101 (12.90)	552 (9.98)	< 0.001
Hospital ownership			< 0.001
Public	29,237 (26.75)	1844 (33.34)	
Private not-for-profit	65,813 (60.22)	2806 (50.73)	
Private for-profit	14,244 (13.03)	881 (15.93)	

Values are expressed as numbers, with percentages in parentheses unless otherwise specified

AIS, acute ischemic stroke; DNR/DNI, do-not-resuscitate orders in patients not intubated; GCS (E + M), eyes and motor score on the Glasgow Coma Scale; ICH, intracerebral hemorrhage; NIHSS, National Institute of Health Stroke Scale; SAH, subarachnoid hemorrhage

^*^For continuous variables, each cell reports the mean ± standard deviation. ^†^For ordinal variables, each cell reports the median value, with the interquartile range in parentheses

**Fig. 2:**
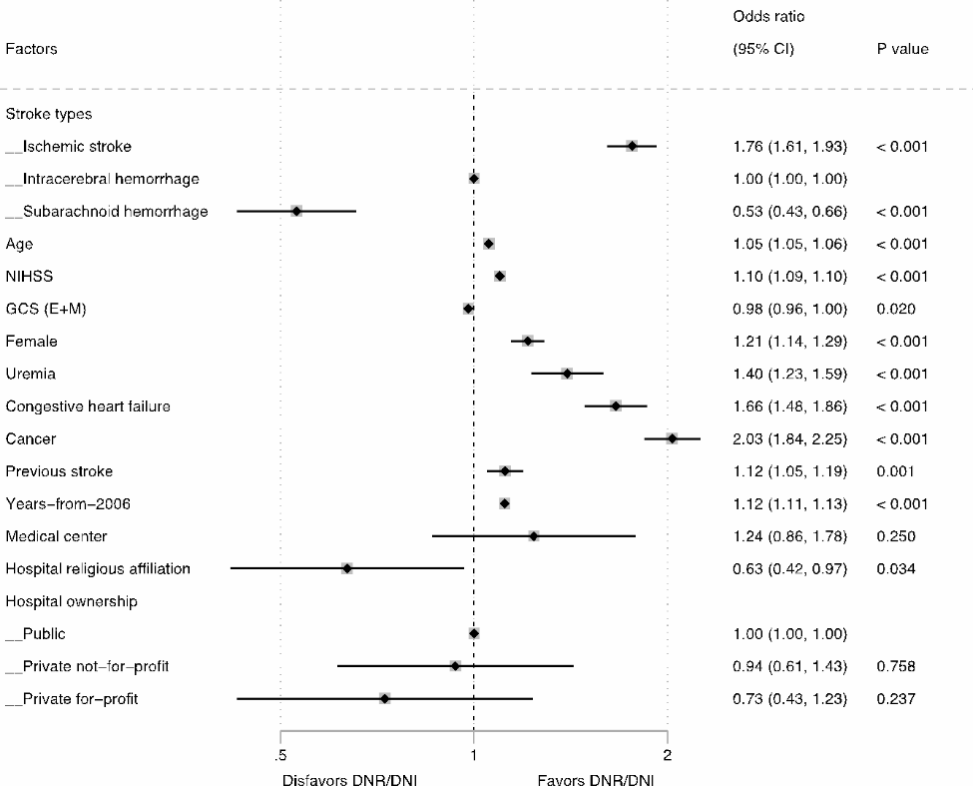
Two-level random effects model for characteristics associated with DNR/DNI use in patients with stroke. CI, confidence interval; DNR/DNI, do-not-resuscitate orders in patients not intubated; GCS (E + M), eyes and motor score on the Glasgow Coma Scale; NIHSS, National Institute of Health Stroke Scale

After adjusting for all other characteristics, patients with AIS were found to be significantly more likely to have DNR/DNI orders (aOR 1.76, 95% CI 1.61–1.93) compared to patients with ICH. On the other hand, patients with SAH were significantly less likely to be associated with DNR/DNI (aOR 0.53, 95% CI 0.43–0.66) compared to patients with ICH (Fig. 2).

**Fig. 3:**
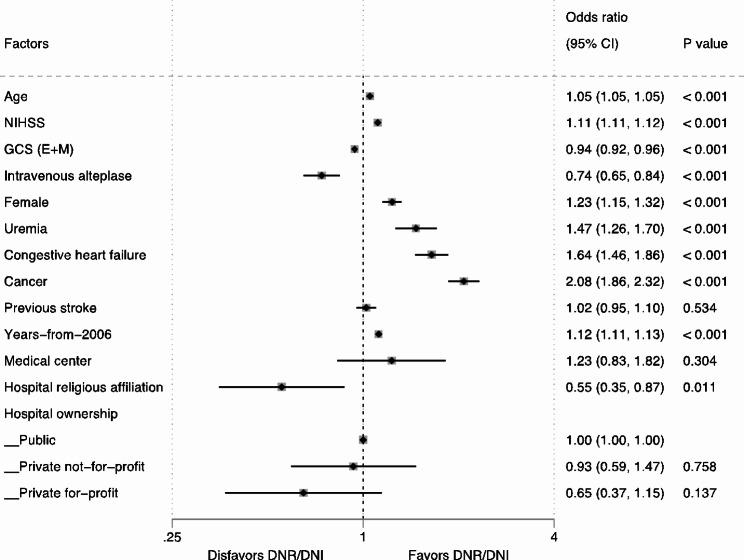
Two-level random effects model for characteristics associated with DNR/DNI use in patients with ischemic stroke. CI, confidence interval; DNR/DNI, do-not-resuscitate orders in patients not intubated; GCS (E + M), eyes and motor score on the Glasgow Coma Scale; NIHSS, National Institute of Health Stroke Scale

**Fig. 4:**
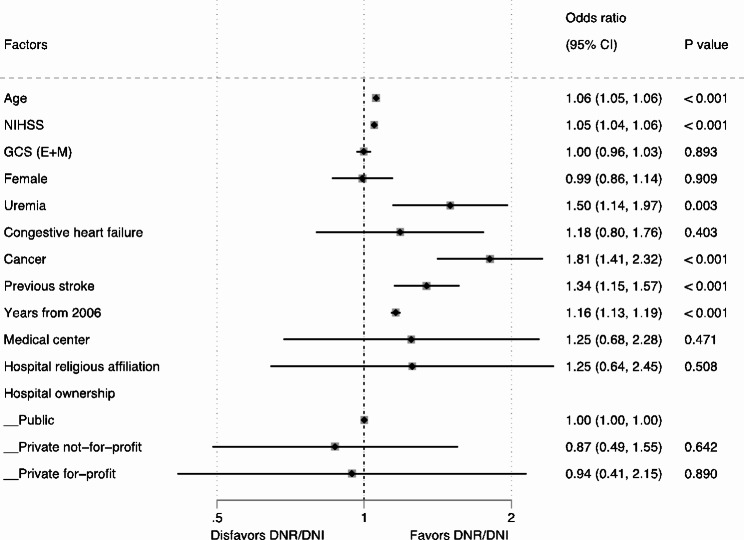
Two-level random effects model for characteristics associated with DNR/DNI use in patients with intracerebral hemorrhage. CI, confidence interval; DNR/DNI, do-not-resuscitate orders in patients not intubated; GCS (E + M), eyes and motor score on the Glasgow Coma Scale; NIHSS, National Institute of Health Stroke Scale

**Fig. 5:**
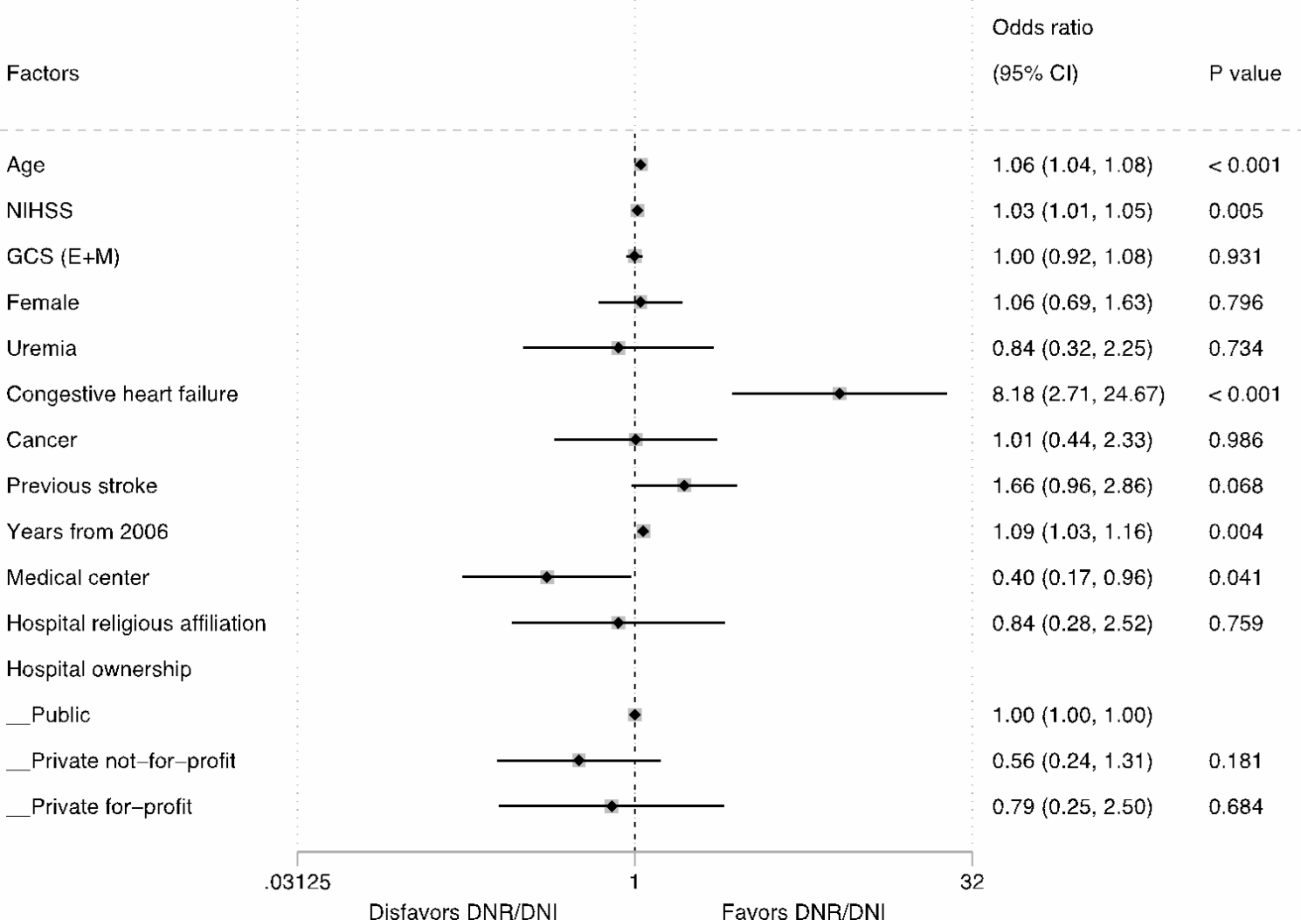
Two-level random effects model for characteristics associated with DNR/DNI use in patients with subarachnoid hemorrhage. CI, confidence interval; DNR/DNI, do-not-resuscitate orders in patients not intubated; GCS (E + M), eyes and motor score on the Glasgow Coma Scale; NIHSS, National Institute of Health Stroke Scale

The overall associations of age (aOR 1.05, 95% CI 1.05–1.06, collectively), NIHSS (aOR 1.10, 95% CI 1.09–1.10, collectively), and years-from-2006 (aOR 1.12, 95% CI 1.11–1.13, collectively) with DNR/DNI were consistent across the three types of stroke (Figs. 2, 3 and 4, and 5). The overall positive associations of uremia (aOR 1.40, 95% CI 1.23–1.59, collectively) and cancer (aOR 2.03, 95% CI 1.84–2.25, collectively) with DNR/DNI were consistent in the subgroups of AIS and ICH (Figs. 2, 3 and 4). Congestive heart failure was positively associated with DNR/DNI in the total population (aOR 1.66, 95% CI 1.48–1.86, collectively) and in the subgroups of AIS and SAH (Figs. 2 and 3, and 5). Females were significantly associated with an increased likelihood of DNR/DNI orders in the total population (aOR 1.21, 95% CI 1.14–1.29, collectively) and the subgroup of AIS (Figs. 2 and 3). Patients with previous stroke were significantly more likely to have DNR/DNI orders in the total population (aOR 1.12, 95% CI 1.11–1.13, collectively) and in the subgroup of ICH (Figs. 2 and 4).

Higher GCS (E + M) scores (aOR 0.94, 95% CI 0.92–0.96) and intravenous alteplase (aOR 0.74, 95% CI 0.65–0.84) were significantly associated with a decreased use of DNR/DNI orders in patients with AIS (Fig. 3).

Patients treated at hospitals with a religious affiliation were significantly less likely to have DNR/DNI orders in the total population (aOR 0.63, 95% CI 0.42–0.97) and in the subgroup of AIS (aOR 0.55, 95% CI 0.35–0.87) (Figs. 2 and 3). Patients with SAH who were cared for at academic medical centers were significantly associated with a lower likelihood of having DNR/DNI orders (aOR 0.40, 95% CI 0.17–0.96) (Fig. 5).

In the sensitivity analyses using complete cases, 97,186 patients had no missing data. Age, NIHSS, and years-from-2006 continued to show positive associations with DNR/DNI orders across all three types of stroke. However, the associations between hospital characteristics and DNR/DNI were no longer significant.

## Discussion

Our study, based on 91,947 patients with AIS, 19,345 patients with ICH, and 3533 patients with SAH, revealed an increase in the proportion of patients with DNR/DNI orders in Taiwan over the past 15 years. According to the Hospice and Palliative Care Act in Taiwan, withholding LST is only allowed for terminal patients, and the definition of “terminal patients” has not changed since the law was enacted in 2000. The increasing proportion of patients with DNR/DNI orders from 2006 to 2020 could be due to several factors, such as an increase in disease severity over time, changing interpretations of the legal definition of terminal patients by physicians, or earlier signing of DNR/DNI consents by patients and their families during the disease trajectory. After adjusting for age, NIHSS, and GCS (E + M), the yearly trend was still significantly increasing, suggesting that the latter two plausible explanations may be more likely. A previous study in a community with a large minority population in the United States reported that early DNR orders (within 24 h) after AIS increased between 2007 and 2016, while it remained stable in patients with ICH [11]. In our study, the proportion of patients with DNR/DNI orders increased in all three types of stroke, which may indicate a gradual general acceptance of end-of-life and palliative care among all stroke patients. The increasing acceptance of palliative care may be due to multiple factors, including legislation, government policy, an aging population, mass media education, the reimbursement of palliative care by the National Health Insurance program, and subsidies for community education provided by hospitals [45, 46]. The practice of end-of-life care in patients with stroke in Taiwan has shifted from beneficence (to avoid death if possible) toward patient autonomy (allowing patients to refuse IMV if they so wish).

In this study, it was found that patients with AIS were more likely to have DNR/DNI orders than patients with ICH, and even more so than patients with SAH. A similar trend was observed in a previous study conducted in Taiwan, where withholding IMV before death was more common in AIS patients than in ICH patients [23]. One plausible explanation is that ICH and SAH are sudden events that require aggressive treatment. In severe cases, patients are intubated early for surgery, and DNR consents are signed only when treatment fails. Although AIS also occurs suddenly and requires timely management with intravenous alteplase or endovascular thrombectomy, anesthesia and IMV are less frequently required than for ICH and SAH. Large hemisphere infarctions and malignant edema often become apparent on the second day or later, allowing more time for shared decision making to avoid IMV during subsequent hospitalization.

Our study found that intravenous alteplase was associated with a significantly lower odds of DNR/DNI orders in AIS patients. Another study also indicated that intravenous alteplase was not significantly associated with an early transition to palliative care [47]. One intuitive explanation may be that as intravenous alteplase is commonly considered as an effective treatment for AIS, it indeed effectively improves patients’ prognosis and reduces the willingness or needs of AIS patients to sign DNR. However, another plausible explanation is that patients who receive intravenous alteplase may be treated more aggressively, which could lead to a lower likelihood of them signing DNR orders.

Previous studies have found that females with AIS have more palliative care encounters [28], a higher incidence of withdrawal of care [31], and greater hospice utilization [30]. Consistent with previous research, our study found that women with AIS were more likely to have DNR/DNI orders than men, even after accounting for age, disease severity, consciousness, and comorbidities. In contrast, although sex differences in the use of DNR orders have been reported in patients with ICH, studies including the present one have shown mixed results [18, 48–50].

Uremia, congestive heart failure, and cancer are among the eight types of non-cancer diseases for which the Taiwan National Health Insurance program has reimbursed for palliative care since 2009. Patients with stroke who also have one of these diseases may be considered terminal patients, as evidenced by the increased use of DNR/DNI orders in this study.

In terms of hospital characteristics, we found a lower likelihood of DNR/DNI orders among patients cared for by hospitals with religious affiliations, both in the total population and the subgroup of AIS. A study of a smaller sample size in the United States, which included a mixed population of AIS and ICH, also showed lower unadjusted DNR rates in hospitals with religious affiliations. However, after adjusting for patient characteristics, the differences disappeared. It is unknown whether the difference would persist if a larger sample size were available [27]. In another study, patients admitted to smaller and for-profit hospitals had lower rates of palliative care use [28]. Additionally, our study found that patients with SAH treated at academic medical centers tend to be less likely to have DNR/DNI orders. Hospital policies and missions can influence the priority setting between beneficence and patient autonomy, which in turn affects physicians’ approaches to discussing end-of-life decisions with patients and their families [32]. All these findings suggest significant influences of hospitals on end-of-life or palliative care decisions among stroke patients.

Some limitations should be noted. First, this study is retrospective and observational, so we could only report associations, not causations. However, we carefully considered the characteristics that preceded DNR/DNI orders in our modeling strategies to reduce possible confounding bias. Second, there is no separate DNI consent form in Taiwan, and the timing of DNR orders is not available in the TSR. Therefore, we could not precisely identify patients with pure DNI orders, nor could we identify “early DNR” in the first 24 h, as in previous studies [11, 12, 15]. Instead, we identified patients with early DNR orders by using DNR without IMV as our primary outcome. Third, the proportion of missing data varied from 0.09% for sex to 11.8% for uremia. We applied the multiple imputation method to eight variables to avoid inefficiency. Still, 1% of patients were excluded because of missing data (Fig. 1). Fourth, laws or guidelines for end-of-life care differ between countries, and the generalizability of the study findings may depend on cultures and behavior patterns in countries. Our study findings may be more generalizable to other countries in Asia than to North America or Europe. Fifth, participation in the TSR is voluntary, so consent bias may be possible. Sixth, although cancer comorbidity is recorded in the Taiwan Stroke Registry, no specific information on type and staging of cancer is available. Seventh, the data in the Taiwan Stroke Registry is reported by professional study nurses after patients are discharged. The reporting of any DNR order is based on the final DNR status recorded in patient’s medical record. Unfortunately, no information is available whether any DNR orders were revoked in these patients and therefore not included in the statistical analysis.

## Conclusions

From 2006 to 2020, the use of DNR/DNI orders increased in all three types of stroke. This trend may indicate a gradual general acceptance of end-of-life care among stroke patients. Furthermore, the study found that hospital characteristics significantly influenced the use of DNR/DNI orders.

### Electronic supplementary material

Below is the link to the electronic supplementary material.

Supplementary Material 1

## Data Availability

The data that support the findings of this study are available from the Taiwan Stroke Registry, but restrictions apply to the availability of these data, which were used under license for the current study, and so are not publicly available. Data are however available from the authors upon reasonable request and with permission of the Taiwan Stroke Registry (taiwanstrokeregistry@gmail.com).

## Abbreviations

AIS: Acute ischemic stroke
aOR: Adjusted odds ratio
CI: Confidence interval
CPR: Cardiopulmonary resuscitation
DNI: Do-not-intubate
DNR: Do-not-resuscitate
GCS (E + M): the combined score of eye and motor tests on the Glasgow Coma Scale
ICH: Intracerebral hemorrhage
IMV: Intubation and mechanical ventilation
LST: Life-sustaining treatment
NIHSS: National Institute of Health Stroke Scale
SAH: Subarachnoid hemorrhage
TSR: Taiwan Stroke Registry
