# Supplementary material for: Patient and hospital characteristics associated with do-not-resuscitate/do-not-intubate orders: a cross-sectional study based on the Taiwan stroke registry
Source: BMC Palliat Care. 2023 Sep 15;22:138. doi: 10.1186/s12904-023-01257-7 (PMC10503153; doi:10.1186/s12904-023-01257-7)
Supplement: Supplementary file 1 — Supplementary Material 1 [file 12904_2023_1257_MOESM1_ESM.docx]

**Taiwan Stroke Registry Investigators**

Li-Ming Lien^2^, Hsu-Ling Yeh^2^, Wei-Hung Chen^2^, Chi-Ieong Lau^2^, Anna Chang^2^, Kuan-Yu Lin^2^, Jiann-Shing Jeng^5^, Sung-Chun Tang^5^, Li-Kai Tsai^5^, Shin-Joe Yeh^5^, Yu Sun^6^, Chien-Jung Lu^6^, Cheng-Huai Lin^6^, Chieh-Cheng Huang^6^, Chang-Hsiu Liu^6^, Hoi-Fong Chan^6^, Chien-Chung Chen^8^, Po-Yen Yeh^8^, Yu-Tai Tsai^8^, Ko-Yi Wang^8^, Yuh-Cherng Guo^12^, Chon-Haw Tsai^12^, Wei-Shih Huang^12^, Chung-Ta Lu^12^, Tzung-Chang Tsai^12^, Chun-Hung Tseng^12^, Kang-Hsu Lin^12^, Woei-Cherng Shyn^12^, Yu-Wan Yang^12^, Yen-Liang Liu^12^, Der-Yang Cho^12^, Chun-Chung Chen^12^, Chung-Hsiang Liu^12^, Cheng-Yu Wei^7,13^, Tzu-Hsuan Huang^13^, Chao-Nan Yang^13^, Chao-Hsien Hung^13^, Ian Shih^13^, Shih-Pin Hsu^14^, Han-Jung Chen^14^, Cheng-Sen Chang^14^, Hung-Chang Kuo^14^, Lian-Hui Lee^14^, Huan-Wen Tsui^14^, Jung-Chi Tsou^14^, Yan-Tang Wang^14^, Yi-Cheng Tai^14^, Kun-Chang Tsai^14^, Yen-Wen Chen^14^, Kan Lu^14^, Po-Chao Liliang^14^, Yu-Tun Tsai^14^, Cheng-Loong Liang^14^, Kuo-Wei Wang^14^, Hao-Kuang Wang^14^, Jui-Sheng Chen^14^, Po-Yuan Chen^14^, Cien-Leong Chye^14^, Wei-Jie Tzeng^14^, Pei-Hua Wu^14^, Chih-Hung Chen^15^, Pi-Shan Sung^15^, Han-Chieh Hsieh^15^, Hui-Chen Su^15^, Ching-Huang Lin^16^, Cheng-Chang Yen^16^, Ruey-Tay Lin^17^, Chun-Hung Chen^17^, Gim-Thean Khor^17^, A-Ching Chao^17^, Hsiu-Fen Lin^17^, Poyin Huang^17^, Huey-Juan Lin^18^, Der-Shin Ke^18^, Chia-Yu Chang^18^, Poh-Shiow Yeh^18^, Kao-Chang Lin^18^, Tain-Junn Cheng^18^, Chih-Ho Chou^18^, Chun-Ming Yang^18^, Hsiu-Chu Shen^18^, An-Chih Chen^19^, Shih-Jei Tsai^19^, Tsong-Ming Lu^19^, Sheng-Ling Kung^19^, Mei-Ju Lee^19^, Hsi-Hsien Chou^19^, Hsin-Yi Chi^20^, Chou-Hsiung Pan^20^, Po-Chi Chan^20^, Min-Hsien Hsu^20^, Wei-Lun Chang^20^, Ya-Ying Wu^20^, Zhi-Zang Huang^20^, Hai-Ming Shoung^20^, Yi-Chen Lo^20^, Fu-Hwa Wang^20^, Ta-Chang Lai^21^, Jiu-Haw Yin^21^, Chung-Jen Wang^21^, Kai-Chen Wang^21^, Li-Mei Chen^21^, Jong-Chyou Denq^21^, Siu-Pak Lee^22^, Ming-Hui Sun^23^, Li-Ying Ke^23^, Po-Lin Chen ^24^, Yu-Shan Lee^24^, Sheng-Feng Sung^25^, Cheung-Ter Ong^25^, Chi-Shun Wu^25^, Yung-Chu Hsu^25^, Yu-Hsiang Su^25^, Ling-Chien Hung^25^, Jiunn-Tay Lee^26^, Jiann-Chyun Lin^26^, Yaw-Don Hsu^26^, Jong-Chyou Denq^26^, Giia-Sheun Peng^26^, Chang-Hung Hsu^26^, Chun-Chieh Lin^26^, Che-Hung Yen^26^, Chun-An Cheng^26^, Yueh-Feng Sung^26^, Yuan-Liang Chen^26^, Ming-Tung Lien^26^, Chung-Hsing Chou^26^, Chia-Chen Liu^26^, Fu-Chi Yang^26^, Yi-Chung Wu^26^, An-Chen Tso^26^, Yu- Hua Lai^26^, Chun-I Chiang^26^, Chia-Kuang Tsai^26^, Meng-Ta Liu^26^, Ying-Che Lin^26^, Yu-Chuan Hsu^26^, Tsuey-Ru Chiang^27^, Mei-Ching Lee^27^, Pai-Hao Huang^27^, Sian-King Lie^27^, Pin-Wen Liao^27^, Jen-Tse Chen^27^, Mu-Chien Sun^28^, Tien-Pao Lai^28^, Wei-Liang Chen^28^, Yen-Chun Chen^28^, Ta-Cheng Chen^28^, Wen-Fu Wang^28^, Kwo-Whei Lee^28^, Chen-Shu Chang^28^, Chien-Hsu Lai^28^, Siao-Ya Shih^28^, Chieh-Sen Chuang^28^, Yen-Yu Chen^28^, Chien-Min Chen^28^, Shinn-Kuang Lin^29^, Yu-Chin Su^29^, Cheng-Lun Hsiao^29^, Fu-Yi Yang^29^, Chih-Yang Liu^29^, Han-Lin Chiang^29^, Chun-Yuan Chang^30^, I-sheng Lin^30^, Chung-Hsien Chien^30^, Yang-Chuang Chang^30^, Ping-Kun Chen^31^, Pai-Yi Chiu^31^, Yu-Jen Hsiao^32^, Chen-Wen Fang^32^, Yu-Wei Chen^33^, Kuo-Ying Lee^33^, Yun-Yu Lin^33^, Chen-Hua Li^33^, Hui-Fen Tsai^33^, Chuan-Fa Hsieh^33^, Chih-Dong Yang^33^, Shiumn-Jen Liaw^33^, How-Chin Liao^33^, Shoou-Jeng Yeh^34^, Ling-Li Wu^34^, Liang-Po Hsieh^34^, Yong-Hui Lee^34^, Chung-Wen Chen^34^, Chih-Shan Hsu^35^,Jian-Jhih Ye^35^, Hao-Yu Zhuang^35^, Yan-Hong Pan^35^, Shin-An Shih^35^, Chin-I Chen^36^, Jia-Ying Sung^36^, Hsing-Yu Weng^36^, Hao-Wen Teng^36^, Jing-Er Lee^36^, Chih-Shan Huang^36^, Shu-Ping Chao^36^, Rey-Yue Yuan^37^, Jau-Jiuan Sheu^37^, Jia-Ming Yu^37^, Chun-Sum Ho^37^, Ting-Chun Lin^37^, Shih-Chieh Yu^38^, Jiunn-Rong Chen^39^, Song-Yen Tsai^39^, Hung-Pin Tseng^40^, Chin-Hsiung Liu^40^, Chun-Liang Lin^40^, Hung-Chih Lin^40^, Pi-Tzu Chen^40^, Chaur-Jong Hu^41^, Nai-Fang Chi^41^, Lung Chan^41^, Chang-Ming Chern^42^, Chun-Jen Lin^42^, Shuu-Jiun Wang^42^, Li-Chi Hsu^42^, Wen-Jang Wong^42^, I-Hui Lee^42^, Der-Jen Yen^42^, Ching-Piao Tsai^42^, Shang-Yeong Kwan^42^, Bing-Wen Soong^42^, Shih-Pin Chen^42^, Kwong-Kum Liao^42^, Kung-Ping Lin^42^, Chien Chen^42^, Din-E Shan^42^, Jong-Ling Fuh^42^, Pei-Ning Wang^42^, Yi-Chung Lee^42^, Yu-Hsiang Yu^42^, Hui-Chi Huang^42^, Jui-Yao Tsai^42^, Ming-Hsiu Wu^43^, Shi-Cheng Chen^43^, Szu-Yi Chiang^43^, Chiung-Yao Wang^43^, Ming-Chin Hsu^44^, Tsang-Shan Chen^45^, Ping-Keung Yip^46^, Vinchi Wang^46^, Shey-Lin Wu^46^, Ching-Kuan Liu^47^, Ryh-Huei Lin^48^, Ching-Hua Chu^48^, Kaw-Chen Wang^48^, Chung-Fen Tsai^48^, Chao-Ching Chen^48^, Chih-Hao Chen^48^, Yi-Chien Liu^48^, Shao-Yuan Chen^48^, Zi-Hao Zhao^48^, Zhi-Peng Wei^48^, Sui-Hing Yan^49^, Yi-Chun Lin^49^, Pei-Yun Chen^49^, Sheng-Huang Hsiao^49^, Bak-Sau Yip^50^, Pei-Chun Tsai^50^, Ping-Chen Chou^50^, Tsam-Ming Kuo^50^, Yi-Chen Lee^50^, Yi-Pin Chiu^50^, Kun-Chang Tsai^50^, Yi-Sheng Liao^51^, Ming-Jun Tsai^52^, Hsin-Yi Kao^52^

^2^Department of Neurology, Shin Kong Wu Ho-Su Memorial Hospital, Taipei, Taiwan

^5^Stroke Center and Department of Neurology, National Taiwan University Hospital, Taipei, Taiwan

^6^Department of Neurology, En Chu Kong Hospital, New Taipei City, Taiwan

^8^Department of Neurology, St. Martin de Porres Hospital, Chiayi County, Taiwan

^12^Department of Neurology, China Medical University Hospital, Taichung, Taiwan

^13^Department of Neurology, Chang Bing Show Chwan Memorial Hospital, Changhua County, Taiwan

^14^Department of Neurology, E-Da Hospital/I-Shou University, Kaohsiung, Taiwan

^15^Department of Neurology, National Cheng Kung University Hospital, Tainan, Taiwan

^16^Department of Neurology, Kaohsiung Veterans General Hospital, Kaohsiung, Taiwan

^17^Department of Neurology, Kaohsiung Medical University Chung-Ho Memorial Hospital, Kaohsiung, Taiwan

^18^Department of Neurology, Chi Mei Medical Center, Tainan, Taiwan

^19^Department of Neurology, Chung Shan Medical University Hospital, Taichung, Taiwan

^20^Department of Neurology, Show Chwan Memorial Hospital, Changhua County, Taiwan

^21^Department of Neurology, Cheng Hsin General Hospital, Taipei, Taiwan

^22^Department of Neurology, Far Eastern Memorial Hospital, New Taipei City, Taiwan

^23^Department of Neurology, Kuang Tien General Hospital, Taichung, Taiwan

^24^Department of Neurology, Taichung Veterans General Hospital, Taichung, Taiwan

^25^Department of Neurology, Ditmanson Medical Foundation Chia-Yi Christian Hospital, Chiayi City, Taiwan

^26^Department of Neurology, Tri-Service General Hospital, Taipei, Taiwan

^27^Department of Neurology, Cathay General Hospital, Taipei, Taiwan

^28^Department of Neurology, Changhua Christian Hospital, Changhua County, Taiwan

^29^Department of Neurology, Taipei Tzuchi Hospital, New Taipei City, Taiwan

^30^Department of Neurology, Min Sheng General Hospital, Taoyuan, Taiwan

^31^Department of Neurology, Lin Shin Hospital, Taichung, Taiwan

^32^Department of Neurology, National Taiwan University Hospital Yunlin Branch, Yunlin County, Taiwan

^33^Department of Neurology, Landseed Hospital, Taoyuan, Taiwan

^34^Department of Neurology, Cheng Ching General Hospital, Taichung, Taiwan

^35^Department of Neurology, China Medical University Beigang Hospital, Yunlin County, Taiwan

^36^Department of Neurology, Taipei Medical University WanFang Hospital, Taipei, Taiwan

^37^Department of Neurology, Taipei Medical University Hospital, Taipei, Taiwan

^38^Department of Neurology, Kuang Tien General Hospital Dajia Division, Taichung, Taiwan

^39^Department of Neurology, Changhua Christian Hospital Yunlin Branch, Yunlin County, Taiwan

^40^Department of Neurology, Lotung Poh Ai Hospital, Yilan County, Taiwan

^41^Department of Neurology, Taipei Medical University-Shuang Ho Hospital, New Taipei City, Taiwan

^42^Department of Neurology, Taipei Veterans General Hospital & National Yang-Ming University School of Medicine, Taipei, Taiwan

^43^Department of Neurology, Chi Mei Medical Center, Liouying, Tainan, Taiwan

^44^Department of Neurology, Buddhist Dalin Tzu Chi General Hospital, Chiayi County, Taiwan

^45^Department of Neurology, Sin-Lau Hospital, Tainan, Taiwan

^46^Department of Neurology, Cardinal Tien Hospital, New Taipei City, Taiwan

^46^Department of Neurology, Yumin Medical Corporation Yumin Hospital, Nantou County, Taiwan

^47^Department of Neurology, Kaohsiung Municipal Hsiao-kang Hospital, Kaohsiung, Taiwan

^48^Department of Neurology, Wei Gong Memorial Hospital, Miaoli County, Taiwan

^49^Department of Neurology, Taipei City Hospital Ren Ai Branch, Taipei, Taiwan

^50^Department of Neurology, National Taiwan University Hospital Hsin-Chu Branch, HsinChu County, Taiwan

^51^Department of Neurology, Taichung Hospital of Health and Welfare Department, Taichung, Taiwan

^52^Department of Neurology, Tainan Municipal An-Nan Hospital-China Medical University, Tainan, Taiwan
